# Endoscopic Ultrasound-Guided Tissue Acquisition Versus Fine Needle Aspiration for Diagnosis of Pancreatic Ductal Adenocarcinoma

**DOI:** 10.7759/cureus.41576

**Published:** 2023-07-08

**Authors:** Tarik W Omairi, Otavio Micelli-Neto, Eloy Taglieri, Jessé C de Araujo-Filho, Andressa Tomé R de Faria, Suzan M Goldman, Rodrigo Cañada T Surjan, Marcel A Machado, Filadélfio E Venco, José C Ardengh

**Affiliations:** 1 Endoscopy, Hospital Moriah, São Paulo, BRA; 2 Endoscopy, Hospital A.C. Camargo Center, São Paulo, BRA; 3 Imaging Diagnostics, Universidade Federal de São Paulo (UNIFESP), São Paulo, BRA; 4 Surgery, Faculdade de Medicina da Universidade de São Paulo, São Paulo, BRA; 5 Surgery, Hospital Nove de Julho, São Paulo, BRA; 6 Gastrointestinal Surgery, Hospital Nove de Julho, São Paulo, BRA; 7 Pathology, Hospital Moriah, São Paulo, BRA; 8 Digestive Endoscopy, Hospital Moriah, São Paulo, BRA

**Keywords:** tissue acquisition, ductal pancreatic adenocarcinoma, histology, pancreatic neoplasms, fine needle aspiration biopsy (fnac), endoscopic ultrasound (eus)

## Abstract

Objectives: Compare the 22G needle versus EchoTip ProCore® 20 (Cook Medical, Bloomington, IN, USA) on their handling, specimen suitability, amount of tissue obtained, diagnostic performance, the possibility of immunohistochemistry, and rate of adverse events.

Materials and methods: This is a retrospective, comparative study of consecutively examined patients with pancreatic masses who underwent endosonography-guided fine needle aspiration (FNA) via the 22G needle, and endosonography-guided tissue acquisition (TA) via ProCore 20 (PC20). The operator evaluated needle insertion and subjectively classified the specimen. The pathologist measured the samples, classified the amount of tissue, and determined the influence of bleeding on the interpretation.

Results: A total of 129 patients participated in the study, out of whom 52 underwent endosonography-guided FNA with 22G and 77 underwent endosonography-guided TA with a PC20 needle. Malignant lesions were found in 106, and 23 had benign lesions. The duodenal route was used in 62% of patients. The 22G needle was easier to introduce (p=0.0495). However, PC20 obtained a larger amount (p<0.01) with fewer punctures (p<0.001). The PC20 also yielded a larger average microcore diameter (p=0.0032). Microhistology was adequate for 22G and PC20 in 22 (42.2%) and 50 (78.1%) specimens, respectively (p<0.001). Bleeding was not significantly different (p>0.999). Immunohistochemistry was possible in 36 (69.2%) and 40 (51.9%) specimens obtained by 22G and PC20, respectively (p=0.075). The sensitivity, specificity, positive predictive value, negative predictive value, and accuracy of 22G were 93.5%, 100%, 100%, 66.7%, and 94.2%, respectively; and for PC20, it was 95%, 100%, 100%, 85%, and 96.1%, respectively. Mild bleeding was the most common early adverse event, occurring in 2/52 (3.8%) 22G and 4/77 (5.2%) PC20 cases (p>0.05).

Conclusions: The PC20 required fewer punctures and reduced the need for immunohistochemistry as it yielded better and larger microcores. Its ease of insertion into the target lesion makes it a good option to obtain satisfactory microcore specimens in difficult positions, such as the transduodenal route.

## Introduction

Endoscopic ultrasound-guided fine needle aspiration (EUS-FNA) has replaced percutaneous and/or surgical biopsy in daily clinical practice and now plays a key role in the diagnosis of pancreatic ductal adenocarcinoma (PDA) [1,2]. This procedure has a sensitivity ranging from 54% to 96%, high specificity, and accuracy between 83% and 95%. However, the conventional 22G and 25G needles used during EUS-FNA have limitations, such as the poor ability to provide histological samples suitable for immunohistochemistry (IHC) or other ancillary methods [3,4].

The development of rapid on-site evaluation (ROSE) with pathologists in the biopsy suite has been shown to improve accuracy [5]. The ROSE is useful during the learning curve, enabling even low-volume centers to achieve >90% accuracy, but its high added cost has made it unfeasible in developing countries [6]. Other issues that have limited its popularization among specialist physicians include the scarcity of trained personnel and the need for specific knowledge among pathologists [7,8].

The race to increase diagnosis rates by obtaining better tissue samples has driven the shift from a cytological approach to a microhistological (McH) one. These McH techniques have been employed since the implementation of EUS-FNA at our facility [9]. Innovation focuses on needle design, which has led to the development of biopsy needles for tissue acquisition (TA) and microcore (MC) retrieval. The EchoTip ProCore® 20 (Cook Medical, Bloomington, IN, USA) needle is designed to balance good flexibility with a large diameter to improve endosonography-guided tissue acquisition (EUS-TA) [10].

The objective of this study was to compare EUS-FNA with a 22G needle versus EUS-TA with the new ProCore 20 (PC20) double forward bevel needle for the diagnosis of PDA in terms of handling, specimen adequacy, the quantity of tissue obtained, diagnostic performance, the possibility of performing ancillary diagnostic methods (IHC), and rate of adverse events (AE).

## Materials and methods

Study design and population

This is a retrospective, comparative, and observational study conducted between January 2016 and December 2020. Adult patients (age >18 years) with suspected solid pancreatic tumors on imaging (abdominal US), CT, or MRI were referred to the Digestive Endoscopy Service of Hospital Moriah, São Paulo, Brazil, for EUS-FNA or EUS-TA-assisted diagnosis. The EUS was performed by experienced physicians (Eloy Taglieri, Otávio Micelli-Neto, and José Celso Ardengh). The study was approved by the Research Ethics Committee of the Federal University of São Paulo (approval no. 5.055.146).

We excluded patients with severe coagulopathy, massive ascites, the absence of an acoustic window for a puncture, cystic lesions identified by EUS, and those who refused EUS after the clarification provided in the informed consent form (ICF), which was presented to and signed by all patients and/or their legal proxies during the study.

Procedure technique

**Figure 1:**
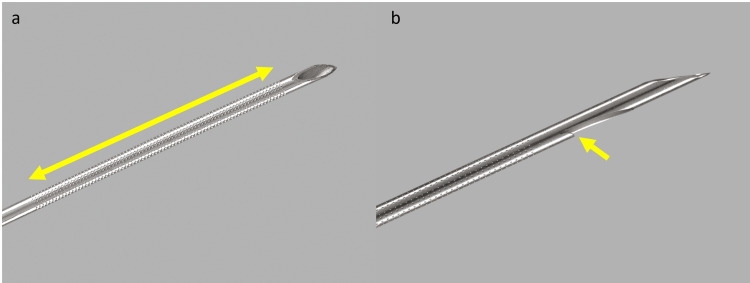
Illustrations of EchoTip® 22G and ProCore® 20 needles, courtesy of Cook Medical a: The 22G needle has tiny fenestrations on the needle body (double-headed yellow arrow) to improve visualization during a EUS examination b: The ProCore® 20 needle tip has an inverted bevel (yellow arrow) to obtain a microcore biopsy EUS: Endoscopic ultrasound

Patients were placed in the left lateral decubitus after the administration of propofol for sedation. All procedures were monitored by an anesthesiologist, and the EUS was performed with Fujinon® EG-530UT and EG-580UT2 linear echoendoscopes (FujiFilm Medical Systems USA Inc., Lexington, MA, USA). During EUS, needle passage was performed after a puncture; with the needle in the center of the target lesion, the stylet was removed, and the needle was submitted back-and-forth, respecting its limits for 10 times (back-and-forth = 1 time) in a fan movement. Suction with a 10 cc syringe was performed at each needle pass, and the stylet was reintroduced into the needle after the first pass and before all punctures in all patients [9]. The EUS-FNA was performed with the EchoTip® 22G needle (Cook Medical, Bloomington, IN, USA), and the EUS-TA was done with the new PC20 needle with a double forward bevel (Figure 1). All tumors of the head and/or unciform process were accessed through the duodenum, while those of the body/tail were accessed through the stomach.

Needle handling

The ease of needle introduction into the tumor was evaluated by the operator as easy or difficult. It was determined to be 'easy' when there was no difficulty in introducing it into the target lesion and 'difficult' when the operator experienced any complication, such as having to reposition the device or unsuccessful attempts to introduce the needle into the target lesion.

Initial specimen evaluation

**Figure 2:**
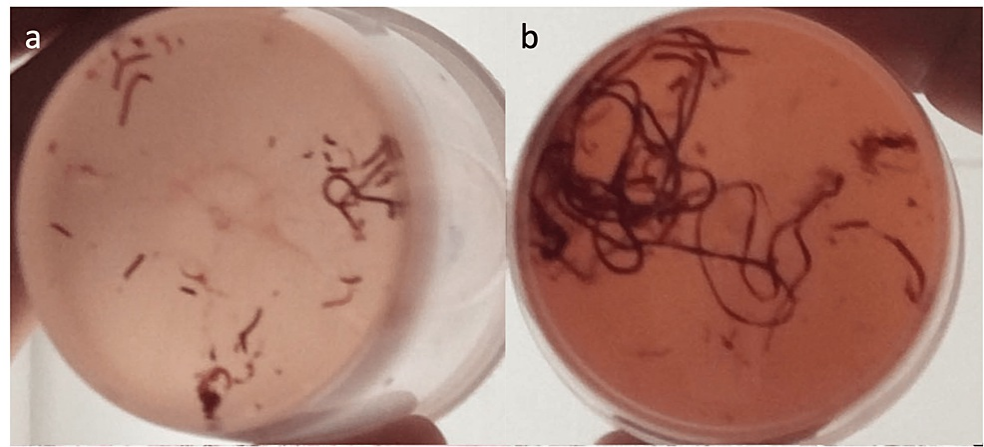
Specimens obtained from the biopsies a: Microcore biopsy obtained by EUS-FNA with a 22G needle; b: Biopsy by EUS-TA taken with a PC20 needle After washing the needles, the collected tissue samples were formalin-fixed (buffered formaldehyde solution at 10%) and processed for pathological diagnosis. EUS: Endoscopic ultrasound, FNA: Fine needle aspiration, TA: Tissue acquisition, PC20: ProCore 20

The amount of tissue obtained was first assessed subjectively by the operator as small (<0.5 cm), medium (0.5 cm to 1 cm), or large (>1 cm fragments) (Figure 2 a, b). Once aspirated, the sample was expelled by fixation in 10% phosphate-buffered formalin. Specimens for McH were obtained from the hub of the 22G needle and the new PC20 by flushing the needle with 2 cc of a 10% buffered neutral formalin solution. Shortly afterward, the stylet was reintroduced into the needle to extract residual content. Specimens were fixed in 10% buffered formalin for six to 24 hours. All were then sent for standard McH processing after paraffin embedding. Any residual fixative contained in the biopsy vials was sent for McH processing (collection of tissue residues and subsequent preparation of tissue blocks by embedding in agarose). All cases were evaluated by a pathologist specializing in pancreatic pathology with more than 25 years of experience (Filadélfio Euclides Venco). The amount of tissue obtained, as well as bleeding, were assessed using objective scores [9]. Smears were prepared and stained per the usual protocol.

Microhistology technique

**Figure 3:**
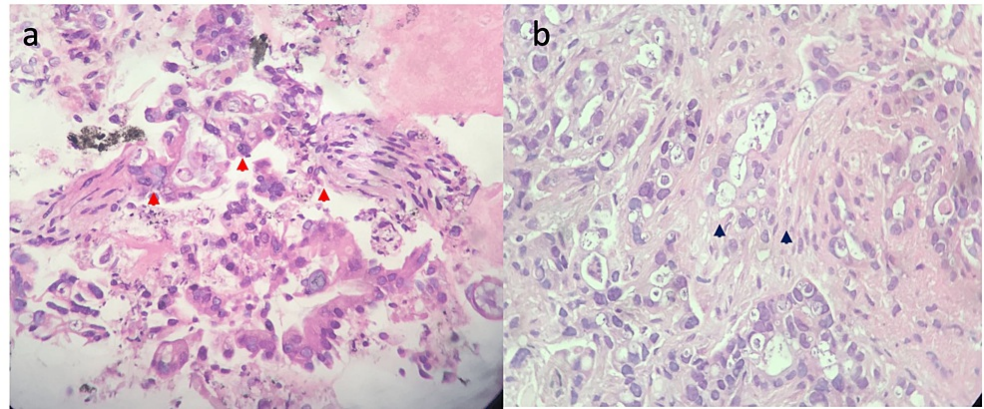
Results of pathological diagnosis a: Clustered atypical pancreatic cells dissociating fibrotic area obtained by the 22G needle (red arrowheads); b: Infiltrating pancreatic adenocarcinoma associated with exuberant desmoplastic reaction (black arrowheads)

The sample obtained was centrifuged for 5 minutes at 2000 rpm. The supernatant was discarded, and MCs were transferred into a 1.5 ml Eppendorf tube, then resuspended in 1 cc of 2% liquid agarose as an intermediate embedding medium. The reaction tube was again centrifuged for 1 minute at 1000 rpm to concentrate the tissue block in the agar. Once solidified, the agar cone with the cells in the top layer was taken out of the reaction tube, wrapped in filter paper, and embedded in paraffin. At this point, the sample could be handled as a routine tissue block. Thin (3 mm) sections from the paraffin-embedded tissue block were cut, mounted on glass slides, and stained with hematoxylin and eosin (H&E). When each slide was examined (Figure 3 a, b), the cellularity, presence of loosely cohesive aggregates or single tumor cells, quality and quantity of cytoplasm, nuclear pleomorphism, chromatin patterns, nucleus-to-cytoplasm ratio, and necrosis were systematically analyzed. If the H&E stains did not provide a clear diagnosis, especially when pancreatic neuroendocrine tumors (p-NET) such as lymphoma and/or sarcoma were suspected, IHC stains were performed using the avidin-biotin-peroxidase method to confirm the diagnosis.

The pathologist measured the size of the MC containing pancreatic and/or neoplastic tissue in millimeters. Pancreatic tissue fragments >0.550 mm were considered MCs. The pathologist then graded the tissue specimen by evaluating the amount of tissue and/or cells on a scale of 1 to 4, where 1 refers to <50 cells; 2 refers to 50 to 100 cells; 3 refers to 100 to 200 cells; and 4 refers to >200 cells. Scores 3 or 4 were considered adequate for McH analysis and obtaining MC [11]. Bleeding was defined as mild (scant hemorrhagic cells), moderate (frequent hemorrhagic cells), or severe (frequent cells and/or visible clots, interfering with histological analysis).

Final diagnosis

The final diagnosis was obtained by integrating the results of EUS-FNA, EUS-TA, and surgery. Follow-up was done after obtaining information from the attending physicians. On a case-by-case basis, the pathologist determined whether there was sufficient material to perform IHC and other ancillary methods.

Adverse events and follow-up

Early AEs (those occurring up to 24 hours after the procedure) were documented at the time of the procedure, while late AEs were recorded through telephone follow-up 24 hours to 10 days after the EUS, which is done as routine at our facility. Clinical, laboratory, and imaging data were included to assist in the identification, stratification, and treatment of AEs.

Statistical analysis

The demographic and clinical characteristics of patients were summarized through medians and ranges (minimum-maximum) for continuous data and through absolute and relative frequencies for categorical data. For comparison of categorical data, the chi-square test, two-proportion z-test, or Fisher’s exact test were used as indicated, while the two-sample t-test or the Mann-Whitney U test was used as appropriate to compare continuous data. Statistical significance was accepted at p<0.05. Datasets were compiled in Microsoft Excel (Microsoft Corp., Redmond, WA, USA), and all statistical analyses were performed in the Microsoft R 3.3.1 software.

## Results

Sample profile

A total of 129 patients with solid pancreatic tumors underwent EUS-FNA (n=52, 40.3%) and EUS-TA (n=77, 59.7%) from January 2016 to January 2017 and February 2017 to December 2020, respectively. Out of which, 67 (52%) were men and 62 (48%) were women, with a mean age of 65 years (range: 25 to 95 years). The 22G needle was used largely in asymptomatic patients (p=0.034), while PC20 was used most often in patients with abdominal pain (p<0.001) and jaundice (p=0.045). These data are shown in Table 1.

**Table 1:** Overall patient characteristics and EUS findings *Chi-square test, **Mann-Whitney U test, ***Proportional test, ****Fisher’s exact test PC20: ProCore 20, EUS: Endoscopic ultrasound, MPD: Main pancreatic duct, FNA: Fine needle aspiration, TA: Tissue acquisition, p-NET: Pancreatic neuroendocrine tumor

Variables	22G: n=52, (40.3%)	PC20: n=77 (59.7%)	P-value
Gender			0.047*
Female	31 (59.6)	31 (40.3)	
Male	21 (40.4)	46 (59.7)	
Age, median (range in years)	67.0 (25-86)	65.5 (32-95)	0.980**
Asymptomatic			0.034*
Yes	31 (59.6)	30 (39)	
No	21 (40.4)	47 (61)	
Symptoms			
Abdominal pain	8 (15.4)	44 (57.1)	< 0.001***
Cholestatic syndrome	9 (17.3)	27 (35.1)	0.045 ***
Diarrhea	3 (5.8)	1 (1.3)	0.358 ***
EUS findings			
Tumor size, median (range in cm)	2.7	2.65	0.9426 **
Dilatation of MPD			0.072*
Yes	16 (30.8)	8 (10.4)	
No	36 (69.2)	69 (89.6)	
Location,			>0.05*
Neck/body	12 (23.1)	22 (28.6)	
Head/uncinate	34 (65.4)	46 (59.7)	
Tail	6 (11.5)	9 (11.7)	
EUS-FNA/TA			< 0.001****
1 pass	19 (36.5)	19 (24.7)	
2 passes	18 (34.6)	56 (72.7)	
>3 passes	15 (28.8)	2 (2.6)	
Immunohistochemistry	36 (69.2)	40 (51.9)	0.075***
EUS results			0.559*
Pancreatic mass	42 (80.8)	67 (8)	
p-NET	10 (19.2)	10 (13)	
Final diagnosis			
Malignant	46 (88.5)	60 (77.9)	0.498*
Adenocarcinoma	25 (48.1)	41 (53.2)	
p-NET	12 (23.1)	11 (14.3)	
Benign	6 (11.5)	17 (22.1)	

Tumor location was similar in the 22G and PC20 groups: 65.4% and 59.7% in the head, 23.1% and 28.6% in the body, and 11.5% and 11.7% in the tail of the pancreas, respectively (p>0.05). There was no significant inter-group difference regarding dilatation of the main pancreatic duct (p=0.07) or mean lesion size (p=0.94), which was 2.7 cm. Most patients (81.4%) did not have bile duct dilatation (as seen in Table 1).

Needle handling and initial specimen assessment

Introduction of the 22G and PC20 needles into the target lesion was difficult in 6 (11.6%) and 12 (15.7%) cases, respectively, with a significant difference (p=0.0495). The number of target lesion punctures required to obtain a satisfactory amount of material when comparing the 22G and PC20 needles was one, two, and three or more punctures in 36.5% and 24.7%, 34.6% and 72.7%, and 28.8% and 2.6% of cases, respectively (p<0.001). These results show that, with the PC20 needle, one (24.7%) or two punctures (72.7%) were needed to obtain high-quality material, while the 22G needle needed more than three punctures to obtain the same result in the majority (63.4%) of patients (Table 1).

According to the operator’s subjective initial assessment, the 22G needle yielded a small, medium, and large specimen in 15.4%, 75%, and 9.6% of cases, respectively, while the PC20 needle yielded no small specimens (0%), medium specimens in 10.3%, and large specimens in 89.7% of cases (p<0.01). Thus, the PC20 needle yielded a significantly larger amount of material (p<0.01) with significantly fewer punctures (p<0.001). Initial subjective evaluation of the material contained in the formalin vial by the physician who performed the EUS showed that the PC20 needle provided a greater amount of material compared to the 22G needle (p<0.001). These results are shown in Table 2.

**Table 2:** Initial assessment by the operator in relation to the tissue sample obtained and the technical difficulty in inserting the needle into the target lesion *Chi-square test EUS: Endoscopic ultrasound, FNA: Fine needle aspiration, TA: Tissue acquisition, PC20: ProCore 20

Needle Type		EUS-FNA (22G), n (%)	EUS-TA (PC20), n (%)	P-value
Specimen acquired	Small	8 (15.4)	0	
	Medium	39 (75)	8 (10.3)	<0.001
	Large	5 (9.6)	69 (89.7)	
Difficulty	Easy	46 (88.8 )	65 (84.3)	
	Difficult	6 (11.6)	12 (15.7)	0.0495

Microhistological and immunohistochemical analysis

**Figure 4:**
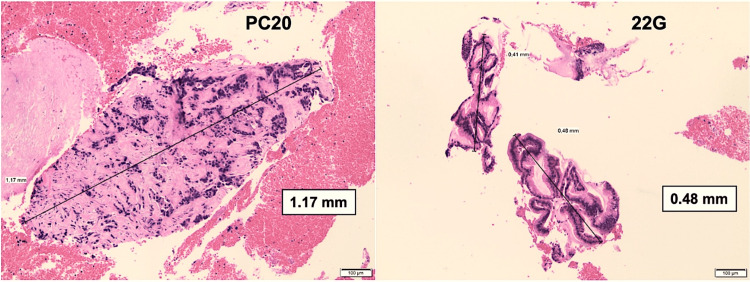
Pancreatic ductal adenocarcinoma PC20: A TA measuring 1.17 mm (black arrow) shows malignant microtubular structures infiltrating the fibrotic area (H&E, 100x) 22G: Collected tissue measuring 0.48 mm (black arrows) shows typical epithelial clusters (H&E, 100x) PC20: ProCore 20, TA: Tissue acquisition, H&E: Hematoxylin and eosin

**Figure 5:**
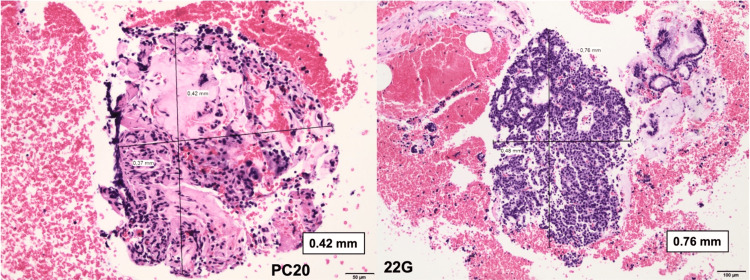
Pancreatic neuroendocrine tumor grade 1 as defined by WHO PC20: A needle TA measuring 0.42 mm (black arrows) shows a duct-insular subtype, with hyalinized stroma (or amyloid-like) (H&E, 50x) 22G: Needle tissue shows small cells with pale pink cytoplasm and round eccentrically located nuclei (H&E, 100x) PC20: ProCore 20, TA: Tissue acquisition, H&E: Hematoxylin and eosin

The PC20 needle provided larger tissue samples when compared to the 22G needle. The mean diameter of the largest specimen (MC) containing apparent lesion tissue obtained by the 22G and PC20 needles was 0.94 ± 0.55 mm and 1.51 ± 1.3 mm, respectively (p=0.0032). Figures 4-5 present a histological comparison of specimens obtained with each needle.

Regarding tissue samples, the 22G needle compared to the PC20 needle yielded specimens graded by the pathologist as 1, 2, 3, and 4 in 17 (32.8%), 1 (1.2%), 13 (25%), and 16 (20.7%) cases, versus 12 (23%), 19 (24.6%), 10 (19.2%), and 41 (53.5%) cases, respectively (p<0.001) (Table 3). Considering that only scores 3 and 4 are suitable for McH analysis and classificatory diagnosis, the 22G and PC20 needles yielded suitable specimens in 22 (42.2%) and 50 (78.1%) cases, respectively (p<0.001). The degree of bleeding identified in the samples by the pathologist was not significantly different, considering the worst performance of each of the needles (p>0.999).

**Table 3:** Comparison of needles in terms of specimen obtained and bleeding *Chi-square test EUS: Endoscopic ultrasound, FNA: Fine needle aspiration, TA: Tissue acquisition, PC20: ProCore 20

Needle type		EUS-FNA (22G), n (%)	EUS-TA (PC20), n (%)	P-value
Specimen acquired	<50 cells	17 (32.8)	1 (1.2)	<0.001
	50 to 100 cells	13 (25)	16 (20.7)	
	100 to 200 cells	12 (23)	19 (24.6)	
	>200 cells	10 (19.2)	41 (53.5)	
Bleeding	Mild	16 (30.7)	24 (31.1)	>0.999
	Moderate	35 (67.4)	51 (66.4)	
	Severe	1 (1.9)	2 (2.5)	

Final diagnosis

There was no statistically significant difference between needles in terms of benign and malignant lesions (p=0.498). The final diagnosis was malignant in 106 (82.1%) and benign in 23 (17.9%) patients. In cases of malignancy, the final diagnosis obtained by follow-up, EUS-FNA, EUS-TA, and surgery was pancreatic ductal adenocarcinoma (PDAC) (70), pancreatic neuroendocrine tumor (p-NET) (23), metastases (7), non-Hodgkin’s lymphoma (2), solid pseudopapillary neoplasia (2), gastrointestinal stromal tumor (GIST) (1), and sarcoma (1). Among the benign masses, 21 patients had pancreatitis nodules, and 2 patients had autoimmune pancreatitis. The sensitivity, specificity, positive predictive value (PPV), negative predictive value (NPV), and accuracy for the 22G and PC20 needles for the diagnosis of PDAC were 93.5% and 95%, 100% and 100%, 100% and 100%, 66.7% and 85%, and 94.2 and 96.1%, respectively (p=0.642). Immunohistochemistry was indicated in 36 (69.2%) and 40 (51.9%) specimens obtained by the 22G and PC20 needles, respectively (p=0.075). There was no significant difference in diagnosis or indication of IHC.

Adverse events

The only early AE that occurred was mild, self-limited bleeding from the wall of the gastrointestinal tract at the puncture site without clinical repercussions, identified in 2/52 (3.8%) cases after puncture with the 22G needle and 4/77 (5.2%) cases after puncture with the PC20 needle (p>0.05). There were no serious late AEs requiring clinical treatment or hospital admission.

## Discussion

During McH analysis of PDA, tissue morphology and architecture are essential for diagnosis, so obtaining an MC is preferable to obtaining cells through 22G and 25G needles [12]. A EUS-TA can establish the diagnosis of PDA, causing a direct impact by indicating neoadjuvant and less aggressive endoscopic procedures, avoiding unnecessary surgical intervention [10,13]. The sensitivity, specificity, PPV and NPV, and accuracy for the diagnosis of PDA with a 22G needle have been reported as 78.4%, 99.2%, 99.3%, 77.2%, and 87.2%, respectively [13]. In our study, these parameters were 93.5%, 100%, 100%, 66.7%, and 94.2%, with no statistical difference versus the PC20 needle (p=0.642). This can be attributed to the preparation of the material for McH and our pathologist’s experience. Larger-gauge needles (19G) obtain more tissue but are difficult to handle and can be associated with an increased frequency of AEs. In this case, the limiting factor is the rigidity of the needle, which makes it difficult to obtain samples from tumors located in the head of the pancreas [14].

To overcome these issues, the EchoTip ProCore® 20G biopsy needle was designed. It combines a double forward bevel, good flexibility, and a larger gauge, allowing MC to be obtained from pancreatic masses. This needle seems to obtain MCs with greater quantity and better quality compared to traditional needles such as the EchoTip 22G and 25G (seen in Figure 2), thus allowing the diagnosis of benign and malignant tumors [12,15]. The MC specimens allow for additional IHC and/or molecular analysis if needed. This technique can distinguish PDA from chronic pancreatitis and diagnose p-NET, autoimmune pancreatitis, lymphoma, or solid groove pancreatitis, all of which mimic PDAC [15-17].

Currently, molecular analysis is increasingly indicated, and obtaining MC is crucial so that these tests can be performed on a single sample. These analyses can be performed on histological and/or cytological samples, but whether the MCs obtained by the EUS-TA are more reliable than those acquired by FNA remains a matter of debate [10]. This phenomenon was observed in our sample.

A recent multicenter case series on PC20 in pancreatic (62.2%) and extra-pancreatic tumors reported adequacy, accuracy, sensitivity, and specificity of 92.4%, 91.5%, 90.8%, and 100%, respectively, for pancreatic lesions [17]. Another study compared the effectiveness of PC20 with the former ProCore® 22G (PC22) and Acquire® (Boston Scientific, Marlborough, MA, USA) 22G (AC22) for diagnosing PDA in 191 patients [18]. Sensitivity was 96.4% with PC20, 58.8% with PC22, and 75% with AC22, with one case of acute pancreatitis occurring after the use of the PC20 needle. The authors concluded that PC20 had greater diagnostic capacity than PC22. Both PC20 and AC22 had similar accuracy, but the sensitivity of PC20 was higher for lesions in which histological evaluation is essential due to the superior quality of the MC obtained [18]. This occurred in our study, where the sensitivity, specificity, PPV, NPV, and accuracy for diagnosing a PDA with PC20 were 95%, 100%, 100%, 85%, and 96.1%, respectively, which is higher than previously reported elsewhere.

Another prospective, randomized study of solid pancreatic tumors revealed that PC20 provided more suitable MCs (p=0.002), especially with a greater diameter (p=0.032) than a 22G needle [10]. Although there was no difference in the final diagnosis between the two, these properties do improve the pathologist’s performance [12]. The old PC22 needle showed a sensitivity of 80% for pancreatic lesions <2 cm. For those with a mean diameter of 32 mm, the sensitivity was 87.5% with a single puncture [18], and 97.8% with two punctures [19]. In our study, the PC20 needle reached a sensitivity of 95% versus 93.5% for the 22G needle (p=0.642). These results can be explained by its larger caliber, which allows fewer punctures, and by the forward bevel design, which yields longer MCs. There was no statistical difference in diagnosis between needle types. This explains the high sensitivity and accuracy of the 22G needle. However, as this study was conducted at a single center at different times with the same specialist pathologist, this constitutes a source of bias. The advantage of PC20 would be to obtain more consistent MCs rather than cells with greater sensitivity, as well as reduce the number of punctures; this would be particularly helpful in low-volume centers, where a dedicated pathologist is often not available for ROSE. It is an unequivocal fact that MCs are easier to interpret than cytology samples by pathologists with no experience in pancreatic cytology. This was confirmed in a study by Novis et al. comparing the results of cytology obtained by ERCP and EUS-FNA in patients with biliary strictures and analyzed by specialist and non-specialist pathologists [20].

The relationship between needle gauge and the quality of tissue specimens obtained has been demonstrated in studies involving 19G needles [21,22]. The old ProCore® 19G was shown to yield good tissue samples with increased accuracy in a study with heterogeneous indications [21]. However, it has not been widely used, and only one study to date has been published, suggesting that its limitations make it an uninteresting choice for this purpose [23]. The difficulty of using this needle in the duodenum was confirmed by another recent prospective multicenter study employing a flexible 19G needle. The authors concluded that it should not be recommended for routine transduodenal use [24]. In our study, when comparing the introduction of the needle into the target lesion, the PC20 needle was more difficult to introduce into target lesions in the unciform process due to its caliber (p=0.0495). However, this did not prevent specimens from being obtained from all patients.

Karsenti et al. compared TA via AC22 to the PC20 and found no significant difference between them in terms of accuracy, but the largest samples were obtained with the PC20 [25]. While the difference could be due to the larger caliber of the needle, it also represents the increased ability of the TA to obtain more MCs.

There was no statistical difference regarding sensitivity, specificity, PPV, NPV, or accuracy. We raise two hypotheses to explain this fact. First, our pathologist has great experience with pancreatic tumors McH, increasing the sensitivity of 22G, and reducing the difference between needles; thus, our findings may not be replicable in centers with less experienced pathologists. Second, the small sample size may have prevented us from demonstrating a true difference between the two, which would constitute a limitation of the study. Although there was no statistical difference in terms of diagnostic accuracy, it should be noted that specimen adequacy, number of cells, and material diameter were all superior with PC20. In this sense, van Riet et al. demonstrated in a recent study that TA promotes greater consensus regarding diagnosis among experienced and relatively inexperienced pathologists, suggesting that TA has greater diagnostic reliability in centers with less experienced pathologists [26].

Another innovation of the PC20 is the presence of a side bevel with a forward-facing (anterograde) cutting edge. This allows for larger tissue specimens compared to 22G, as it collects tissue while pushing the needle forward into the target lesion, which is the most effective motion in EUS-TA. In our study, the PC20 needle yielded specimens with a larger mean diameter (p=0.0032); 22 (42.2%) of those obtained with 22G versus 50 (78.1%) obtained with PC20 were suitable for McH (p<0.001). A recent study also concluded that the forward bevel, which enters and exits the target lesion, can cut the surface of the tumor more effectively than the needle tip within the tumor [27]. We consider this undeniable, as we have obtained MC almost twice as large (in mm) as those obtained with the 22G needle.

Regarding MC recovery, data on the new PC20 needle is still scarce. A prospective study involving 53 patients reported 96.2% adequate tissue after a single pass, performed by the slow-pull technique without ROSE [28]. In our study, we were able to obtain 98.8% of tissue specimens in 77 patients after an average of 1.8 punctures performed by the aspiration technique, also without ROSE.

The score used in our study to identify MCs was obtained by grading the tissue sample by evaluating the amount of tissue and/or cells on a scale of 1 to 4, with scores of 3 to 4 considered adequate for McH analysis. An MC was defined as a fragment of intact tissue at least 0.055 mm long on its major axis. This is in agreement with the literature, which reports some similar definitions of MC, e.g., a fragment as large as a high-power microscopic field [18]. This scoring system is simple to use in routine procedures and can easily distinguish between inadequate and adequate tissue samples for cytology. Our results are in agreement with the aforementioned study by Nishioka et al., suggesting greater adequacy for histology with PC20 compared to 22G [29].

Another advantage of the PC20 is that fewer punctures are needed to obtain suitable MCs. In our study, the average number of passes reported for the PC20 was 1.8, significantly less than the 22G, which had an average of 2.4 passes/patient. Therefore, the use of PC20 as a valid alternative to EUS-TA in the transduodenal window is feasible according to recently published European guidelines. Furthermore, AEs were similar when comparing the two types of needles.

Our study has a few limitations. Some have been mentioned above; others are noted below. This was a retrospective study; although data collection was prospective, it was done many years ago and is thus subject to selection bias regarding the type and/or size of the needle. A comparison between the two needles showed no relevant difference in terms of patient age, lesion location, size, or final diagnosis. However, we found a statistically significant difference in relation to sex and the presence or absence of symptoms. The PC20 was used in a greater number of men and predominantly in symptomatic patients (with abdominal pain and jaundice) compared to the 22G, reflecting the retrospective nature of the study. Second, only one pathologist was involved in this study (central analysis). However, tissue specimens were measured by dedicated software, which mitigates this limitation. Third, we found an association between the use of the PC20 needle and fewer punctures needed to obtain an adequate specimen, without ROSE, but with a subjective assessment for which evidence is lacking in the literature.

## Conclusions

The 22G and PC20 needles both achieved satisfactory sensitivity and accuracy. However, the PC20 required fewer punctures, reduced the need for IHC, and obtained a better and larger MC. Due to its ease of insertion into the target lesion, even in difficult positions with the transduodenal route, the PC20 is a good option to obtain satisfactory specimens of MC. In addition to all these advantages, the PC20 obtains a large amount of tissue without the implementation of ROSE by pathologists in the biopsy room, which is a major advantage over other needles dedicated to providing cellular support for diagnosis.
